# The term Wellens syndrome should be extended to include inferior and posterior wall coronary artery disease: a case report

**DOI:** 10.1186/s12245-026-01322-x

**Published:** 2026-07-31

**Authors:** András Simon, Zsófia Nagy, Zoltán Járai

**Affiliations:** 1 Department of Cardiology, South Buda Center Hospital - Szent Imre University Teaching Hospital, Budapest, Hungary; 2 Section of Angiology, Heart and Vascular Center, Semmelweis University, Budapest, Hungary

**Keywords:** Wellens syndrome, Inferior Wellens syndrome, Posterior Wellens syndrome, ECG, Acute coronary syndrome

## Abstract

**Background:**

According to current terminology, Wellens syndrome’s electrocardiographic (ECG) signs are present in leads V2-3 and indicate a critical proximal left anterior descending coronary artery (LAD) stenosis, suggesting an impending anterior myocardial infarction. This case report highlights that the Wellens pattern can be observed in localisations other than the anterior leads, and that the underlying pathomechanism of Wellens syndrome is not specific to the LAD.

**Case presentation:**

An 83-year-old female patient with high cardiovascular risk presented to the emergency department with symptoms characteristic of Wellens syndrome. However, the Wellens pattern appeared in the ECG leads representing the inferior and posterior walls. Urgent coronary angiography confirmed a 99% ostial stenosis of the dominant right coronary artery, which was successfully treated with a drug-eluting stent.

**Conclusions:**

We suggest extending the Wellens syndrome terminology to include inferior and posterior wall coronary artery disease, based on electrocardiographic abnormalities in locations other than the anterior, if the clinical presentation corresponds.

## Background

Current medical literature and guidelines [1] refer to Wellens syndrome when the ECG shows symmetrical, deeply inverted T waves or biphasic positive-negative T waves in leads V2 and V3, with isoelectric or minimally elevated (< 1 mm) ST segments during the patient’s current pain-free state. Further typical features include the absence of precordial Q waves or loss of precordial R waves, a history of angina pectoris, and normal or only mildly elevated serum cardiac biomarkers. The clinical and ECG abnormalities characteristic of Wellens syndrome indicate the complete or near-complete occlusion and spontaneous reopening of the LAD. Previously, two types of Wellens patterns were distinguished: type A and type B. The biphasic T-wave form with positive-negative deflection, type A (Fig. 5, Panel 1, A) was found less often, while the deeply inverted, symmetrical T-wave form, type B (Fig. 5, Panel 1, B) was observed more often [2]. 

In recent years, it has become clear that these two forms evolve into one another over time during spontaneous coronary reperfusion [3]. In the case of occlusion, which may be caused, for example, by vasospasm in rare cases [4] or, more commonly, by thrombus formation on an unstable plaque, ST elevation occurs. This is not always detectable by ECG because if spontaneous recanalisation occurs, the ST-elevation regresses, and symmetrical, deep negative T waves, characteristic of reperfusion, develop in these leads. During the transition between the two, biphasic T waves of positive-negative polarity are detected (Fig. 5, Panel 1).

In Wellens syndrome, a stress test is not recommended, but coronary angiography is indicated as soon as possible because of the critical coronary stenosis threatening a large myocardial mass [5, 6]. 

## Case presentation

The 83-year-old female patient had a medical history of treated hypertension, hyperlipidaemia, chronic renal disease, and left breast ablation for carcinoma. Over six months, she visited the Emergency Department (ED) four times for sudden palpitations and weakness. She complained of chest pain once. The ECGs were interpreted by the medical staff as showing no evidence of acute myocardial ischaemia. The subtle ECG abnormalities observed in the inferior leads were considered nonspecific. Based on the tall, peaked T waves observed in V2-3, the possibility of hyperkalaemia was raised, but laboratory tests consistently ruled it out. Biomarker high-sensitivity troponin I levels were generally within the physiological range, with only one exception of a slightly above-normal value (42 ng/L / normal range: < 13 ng/L). During which ED visit, hypertension was diagnosed as the cause of her complaints, and she was discharged home. Based on the results of the essentially negative 24-hour Holter monitoring and echocardiography, which did not reveal structural heart disease or wall motion abnormality, performed in a cardiology outpatient clinic, her complaints were considered to be of extracardiac origin.

**Fig. 1:**
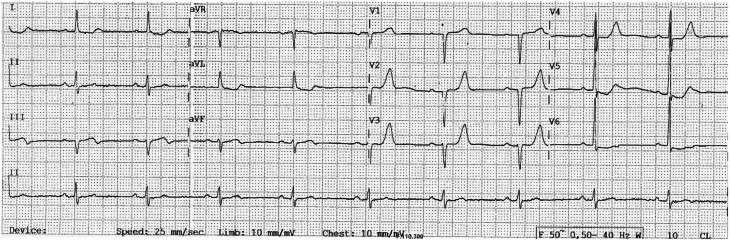
ECG taken on arrival at the Emergency Department

On the fifth occasion, she was admitted to the ED due to sudden weakness and subsequent loss of consciousness in public. On examination, she denied chest pain during or after her fainting episode. The ECG was basically identical to those recorded on previous occasions. (Fig. 1.) Her laboratory results showed high-sensitivity troponin I levels increasing from 86 ng/L to a maximum of 116 ng/L.

**Fig. 2:**
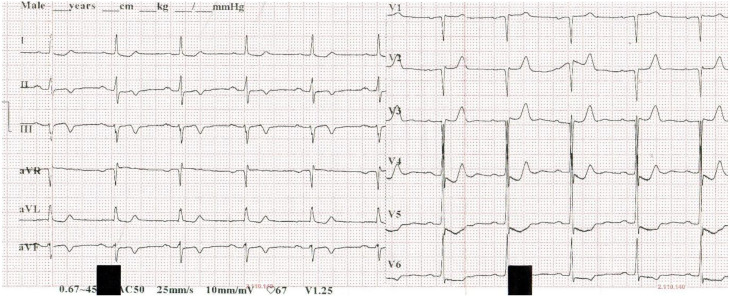
Follow-up ECG performed the next morning

On initial observation in the ED, the patient was administered antihypertensive therapy for elevated blood pressure, acetylsalicylic acid and low molecular weight heparin for suspected acute coronary syndrome, with which she remained symptom-free, and her cardiorespiratory system was stable. Figure 2 shows the ECG taken the next morning in her normotensive, stable, pain-free state.

Echocardiography showed good left ventricular systolic function with no segmental wall motion abnormality and no significant valve abnormality.

**Fig. 3:**
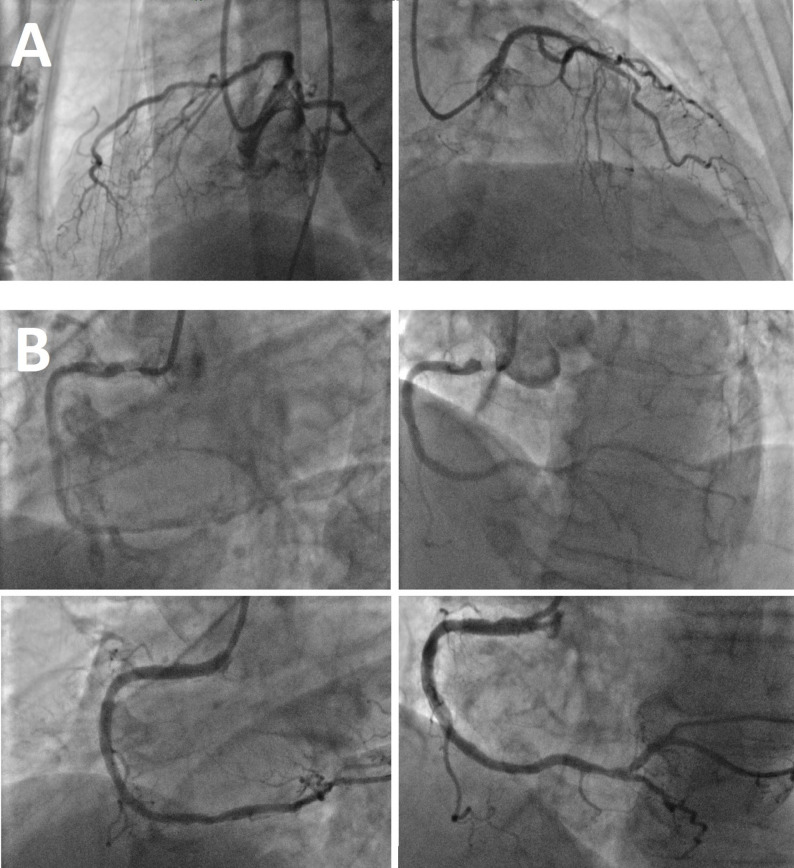
Panel **A**: Left anterior descending coronary artery angiography showed only wall irregularities with a preserved lumen. Panel **B** upper frames: 99% ostial stenosis of the dominant right coronary artery on the coronary angiogram. Panel **B** lower frames: coronary angiogram after the intervention

Nevertheless, based on the positive-negative T-wave morphology in leads II, III, and aVF, and the tall, symmetric, positive T waves in leads V2-3, the cardiologist suspected critical stenosis of the coronary artery supplying the inferior and posterior walls, intermittently leading to occlusion and spontaneous recanalisation. Urgent coronary angiography was initiated. The left coronary artery angiography showed only wall irregularities with a preserved lumen; thus, the angiographic findings could not account for the poor R-wave progression or the T-wave morphologies observed in leads V2–V3 (Fig. 3 Panel A). The right coronary angiography confirmed a 99% ostial stenosis. This artery also supplied the posterior wall. During the successful right coronary intervention with balloon pre- and post-dilatation, one drug-eluting stent was implanted (Fig. 3 Panel B). Subsequently, the patient was discharged home without any complaints.

### Discussion and conclusions

It does not seem reasonable, and no evidence supports that the assumed mechanism of intermittent coronary occlusion and spontaneous reperfusion underlying the Wellens pattern is exclusively related to the LAD and would be expected to present only in the anterior leads. However, its occurrence in other localisations is rarely reported. To our knowledge, to date, two cases of biphasic, positive-negative T waves in the inferior leads, fulfilling other criteria for Wellens syndrome, have been described in peer-reviewed literature. In these cases, critical stenosis was found in the right coronary artery [7, 8]. 

In a study, tall, positive T waves in V2-3 were identified as a hallmark of reperfusion following revascularisation in confirmed posterior wall infarction. With good reason, the authors named this phenomenon posterior wall Wellens syndrome [9]. In such cases, the prominent, high T waves seen in the precordial leads correspond to a mirror image of the symmetrical, deep negative T waves in the posterior wall.

**Fig. 4:**
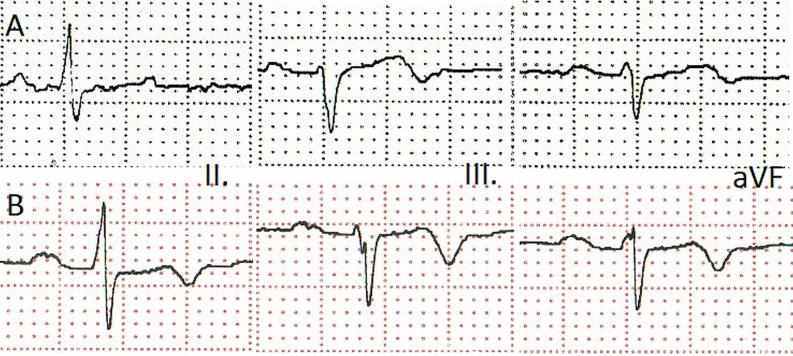
Enlarged beats from the case, showing the Wellens pattern in the inferior leads: in Panel **A**, on the initial recording; in Panel **B**, on the control ECG taken the following day

In inferior Wellens syndrome, T-wave inversion or positive-negative biphasic T waves are observed in the inferior leads (Fig. 4), indicating critical stenosis of the right coronary artery or the circumflex branch, which supplies the inferior wall of the heart.

These ECG abnormalities, if the artery with the critical stenosis also supplies blood to the posterior wall, could be associated with tall, peaked positive or biphasic, negative-positive T waves in the V2-3 leads. From a vector physics perspective, V2–V3 and the posterior wall occupy opposite poles along the horizontal anatomical axis. During posterior reperfusion, the altered repolarisation sequence projects a T-wave vector anteriorly, registering as a prominent positive deflection that mirrors the classic anterior Wellens pattern.

**Fig. 5:**
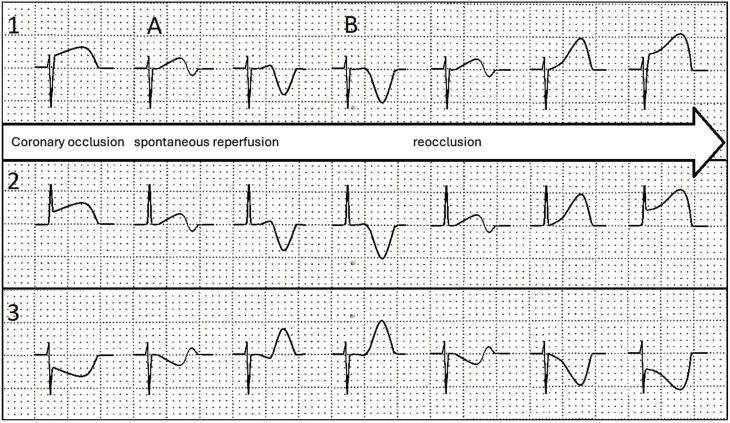
Schematic illustration of ECG changes in Wellens syndrome. Panel 1 shows ST elevation in precordial leads in the case of occlusion of the coronary artery supplying the anterior wall. With spontaneous reperfusion, ST elevation first regresses, and the T wave becomes biphasic, positive-negative, which is known as Wellens syndrome type A (**A**). This can later gradually develop into Wellens syndrome type B (**B**), characterised by deep, symmetrical T-wave inversion. In the event of coronary reocclusion, type B reverts to type A, and ST elevation reappears after ECG pseudonormalisation. Panel 2 shows a schematic view of the same phenomenon in the inferior leads when the coronary artery supplying the inferior wall is involved. Panel 3 shows a schematic view of the same process in the precordial leads when the coronary artery supplying the posterior wall is involved

In patients presenting with angina-like symptoms or myocardial ischaemia, recognising new-onset symmetric T-wave inversion or biphasic positive-negative T-waves in the anterior (Fig. 5, Panel 1) or inferior leads (Fig. 4, Panels A and B; Fig. 5, Panel 2) is crucial, as they may indicate a critical coronary stenosis or a pre-occlusive condition. In this case—although the patient had a history of angina—there was no typical angina prior to the index event, which made it difficult to properly assess the symptoms; this illustrates that a Wellens-like pattern should be considered as a red flag even in the absence of typical chest pain. When the coronary artery supplying the posterior wall is subject to intermittent occlusion and reopening, as described above, negative-positive or high-peaked, symmetric, positive T waves are seen as mirror-image phenomena in the precordial leads (Fig. 5, Panel 3). Even in the presence of stable haemodynamics and a pain-free state, patients with these ECG abnormalities require an early invasive strategy and, if possible, coronary intervention to prevent potentially fatal myocardial infarction.

Spontaneous reperfusion following transient coronary occlusion associated with critical proximal atherosclerotic stenosis or vasospasm is characteristic not only of the left anterior descending artery but may also occur in the coronary arteries supplying the inferior or posterior wall, resulting in distinctive ECG patterns. Therefore, if the clinical presentation is consistent with Wellens syndrome, we propose expanding its definition to include non-LAD coronary arteries. Alternatively, if Wellens syndrome remains strictly defined by LAD disease, we suggest introducing the terms ‘inferior Wellens syndrome’ and ‘posterior Wellens syndrome’ for these locations, both carrying a recommendation for early invasive management.

## Data Availability

No datasets were generated or analysed during the current study.

## Abbreviations

ECG: Electrocardiogram, electrocardiographic
ED: Emergency department
LAD: Left anterior descending coronary artery
